# Foley catheter for cervical priming in induction of labour at University Obstetrics Unit, Colombo, Sri Lanka: a clinical audit with a patient satisfaction survey

**DOI:** 10.1186/s13104-017-2478-z

**Published:** 2017-04-12

**Authors:** M. Patabendige, A. Jayawardane

**Affiliations:** 1 University Obstetrics Unit, De Soysa Hospital for Women, Colombo-08, Colombo, Sri Lanka; 2 Department of Obstetrics and Gynaecology, Faculty of Medicine, University of Colombo, Kinsey Road, Colombo, Sri Lanka

**Keywords:** Induction of labour, Mechanical ripening, Foley catheter, Audit, Sri Lanka

## Abstract

**Background:**

Intracervical insertion of a Foley catheter (FC) has shown to be a safe, effective and relatively feasible mechanical method of cervical priming in induction of labour (IOL). We evaluated indications, effectiveness, patient acceptability and outcomes of FC use in IOL adhering to the ward protocol in our unit.

**Methods:**

A clinical audit with a patient satisfaction survey conducted between July and September 2013 in University Obstetric Unit, Colombo, Sri Lanka. Patients selected for IOL for obstetric reasons were primed with Foley as per ward protocol. All had singleton pregnancies with cephalic presentation, intact membranes and period of gestation of 37 weeks or above. Women with a history of more than one caesarean section or uterine surgery, low-lying placenta and fetal growth restriction were excluded. Subjects who had a Modified Bishop Score (MBS) of less than 3, a 16Fr FC was inserted into cervical canal. Catheter was left undisturbed until spontaneous expulsion or no longer than 48 h. In women with MBS of less than 6 at 48 h after FC insertion, 3 mg prostaglandin E2 vaginal tablet was used subsequently. Artificial membrane rupture with or without oxytocin was used if MBS of 6 or more and in women not in labour 24 h after prostaglandins. Patient satisfaction for Foley insertion was assessed with regards to the degree of comfort using a validated visual analogue scale (0–10).

**Results:**

There were a total of 910 deliveries during the study period. Fifty-six women were primed with FC. Thirty-two (57%) were nulliparous. During induction of labour, 53(95%) reported mild or no discomfort. MBS of 6 or more was achieved in 36/56 (64%) Foley insertions. Twenty needed further intervention with prostaglandins. FC only group had 5 caesarean sections and 31 vaginal deliveries and Foley/prostaglandin group had 7 caesarean sections and 13 vaginal deliveries. Of the 24 women who were induced due to completion of 41 weeks of gestation with otherwise uncomplicated pregnancies, 17 had MBS >6 post priming with Foley and 20 (83%) delivered vaginally. Subjects who had Foley only had a lesser chance of having a caesarean delivery compared to subjects who had Foley followed by prostaglandin (relative risk = 0.40, 95% CI = 0.15–1.09, P = 0.09).

**Discussion:**

FC is a good choice for pre-induction cervical priming with high patient comfort. FC becomes more important in IOL cost reduction in our setting. FC alone seems to be an effective for IOL in women who have completed 41 weeks of gestation with otherwise uncomplicated pregnancies.

## Background

Induction of labour (IOL) is defined as the process of artificially stimulating the uterus to initiate labour [1]. IOL is common obstetric procedure with rising rates worldwide. Sri Lanka has a rate of IOL of 37.5%, which is one of the highest rates in the world [2]. In the United Kingdom, the rate of IOL ranges from 6 to 25% with the average being about 20% [3]. In USA the average rate of IOL is approximately 13% [4]. IOL has a significant impact on the birth experience of women. IOL is indicated if benefits of delivery outweigh the risk of continuing pregnancy.

Different methods are used for IOL in women with an unfavourable cervix. Mechanical methods such as transcervical extra-amniotic Foley catheter (FC) insertion and pharmacological methods such as vaginal prostaglandin E2 and misoprostol are used for IOL in women with an unfavourable cervix for pre induction cervical priming [5–7]. Mechanical methods of induction were developed to promote cervical ripening and the onset of labour by stretching the cervix. They are amongst the oldest methods to initiate labour [8]. Recently mechanical methods have been replaced by pharmacological methods [8]. Readiness of the cervix for onset of labour is assessed by the Modified Bishop Score (MBS) which takes into account the consistency and dilation of the cervix, the station and position of the presenting part [9].

Recent Cochrane review has concluded that IOL using mechanical methods such as FC results in similar caesarean section rates to prostaglandins and yields a lower risk of hyperstimulation with or without fetal heart rate changes compared to prostaglandins [8]. FC gives fewer maternal and neonatal side-effects in comparison with vaginal prostaglandins [10]. When compared with oxytocin, mechanical methods reduce the risk of caesarean section [8]. Mechanical methods are as effective in achieving delivery within 24 h of intervention as any prostaglandins [8]. In terms of caesarean section, they are equally effective and have less side effects [7, 8, 10]. According to the limited data available, there is no evidence of an increased risk of infectious morbidity with mechanical methods [8]. FC for cervical ripening is a far cheaper option to prostaglandin or oxytocin in terms of medication/device cost. The latter methods also incur significant additional cost in monitoring the maternal and fetal wellbeing during the process. Therefore FC is a logical option to consider in limited-resource settings with relative lack of monitoring facilities. Other potential advantages of mechanical methods over pharmacological ones may include wide availability and reduction of some of the side effects [8].

Prostaglandin preparations used for cervical ripening are expensive and unstable, requiring refrigerated storage [11, 12]. In a Nigerian study, feasibility to use prostaglandins was found to be limited largely due to its higher cost and inadequate infrastructure to maintain the narrow temperature range to keep its potency [13]. In experienced hands it is a safe and reliable method. FC for cervical ripening is an obvious choice but many practitioners find it cumbersome, somewhat archaic and esthetically suboptimal with low patient acceptability. Patient preference however is determined by many factors and variable among populations.

FC for IOL is a well-researched topic in published literature. However, data on patient satisfaction or patient preferences in IOL are sparse [8]. One study reported on patient satisfaction and discomfort associated with insertion and cervical ripening in single balloon catheter, prostaglandins and double balloon catheter using a visual analogue scale [7]. In designing the study, we focused more on acceptability and local applicability of FC in our unit. We conducted a prospective audit at a University Obstetric Unit in a major Teaching Hospital in Sri Lanka. This audit was conducted to evaluate the indications, effectiveness, patient acceptability and outcomes of FC in pre-induction cervical priming.

## Methods

A clinical audit with a patient satisfaction survey conducted prospectively between July and September 2013 in University Obstetric Unit, De Soya Hospital for Women (DSHW), Colombo, Sri Lanka. Before embarking into this audit our main cervical ripening method was vaginal prostaglandin E2. Women selected for IOL for Obstetric reasons were offered IOL with FC according to the ward protocol as described below. This ward protocol was developed according to the latest guideline on IOL published by Sri Lanka College of Obstetricians and Gynaecologists (SLCOG) [14].

Ward protocol for cervical priming with FC.

1. Decision for IOL was made by a consultant obstetrician considering obstetric necessity.
2. In women who had a MBS of less than 3, a 16Fr FC was inserted under aseptic conditions into cervical canal, position confirmed with ultrasound and balloon inflated with 50 ml of water.
3. The catheter was left undisturbed until spontaneous expulsion or no longer than 48 h. Regular tugging on catheter was performed by midwives 12 hourly.
4. At 48 h, women were reassessed. If MBS <6, prostaglandin E2 vaginal tablets (maximum two doses of 3 mg 12 h apart for primip and 3 mg single dose for multip) were used subsequently for cervical ripening.
5. If MBS >6 at 48 h and woman was not in labour, artificial membrane rupture and oxytocin was used as per guideline [14].

The selection of patients for FC was not affected by this study. We prospectively audited how FC use occurred over a 3 month period. All the women had singleton pregnancies with cephalic presentation, intact membranes and period of gestation of 37 weeks or above at the time of recruitment. However, women with a history of more than one caesarean section or previous uterine surgery, low-lying placenta and fetal growth restriction were excluded from the study. None of the women approached, declined participation. Once decision for IOL was made, the study was introduced to the eligible women and consecutive consenting study participants were included in the study. MBS in all cases were assessed and all the FCs were inserted by a medical officer trained in the technique.

Data on indications for IOL, duration of Foley in situ, insertion of additional prostaglandins and whether the artificial membrane rupture and oxytocin used or not, outcome of IOL (vaginal delivery or caesarean section), degree of discomfort as assessed by visual analogue scale, duration of labour and maternal and fetal/neonatal complications were obtained. Patient satisfaction for Foley insertion was assessed with regards to the degree of discomfort using a validated visual analogue scale (0–10) by an independent assessor. Visual analogue scale is a simple assessment tool consisting of a 10-point line with 0 on one end, representing no discomfort, and 10 on the other, representing the worst pain ever experienced. In this scale 1, 2, 3 for mild discomfort, 4, 5, 6 for moderate discomfort, 7 or more for severe discomfort were used when interpreting the degree of discomfort. Data analysis was done using standard statistical methods. Fisher’s Exact Test was performed for significant testing among categorical variables (having a MBS of 6 or more and duration of Foley in situ up to 48 h). P value <0.05 was considered as statistically significant.

## Results

Our unit had a total of 910 deliveries during the study period. Fifty-six consecutive cases which were primed with FC, prospectively recruited for the study over 3 months. There were 45 cases of non-eligible women undergoing labour induction by methods other than FC as first choice. Informed consent was given by all of them and therefore, 56 cases were studied. According to Table 1, 32 (57%) were nulliparous, 48 (86%) were between 21 and 35 years of age. Gestational diabetes and women who have completed 41 weeks of gestation with otherwise uncomplicated pregnancies were the commonest indications (Table 3) for IOL. Mean (SD) duration of FC in situ was 31.4 (16.04) h.

**Table 1:** Demographic details of the study participants

Study participants	n = 56, n (%)
Parity	
Parity-0	32 (57)
Parity-1	15 (27)
Parity-2	5 (9)
Parity-3	3 (5)
Parity-4	1 (2)
Period of gestation (weeks)	
37–40 + 6 days	32 (57)
≥41	24 (43)
Age (years)	
≤20	6 (11)
21–25	12 (21)
26–30	22 (39)
31–35	14 (25)
>35	2 (4)

MBS of 6 or more was achieved in 36 (64%) subjects with Foley insertions out of 56. Rest of the 20 (36%) needed further intervention with vaginal prostaglandin E2. As presented in Table 2, 36 out of all (64%) had cervical priming only with FC and 31 of them (31/36, 86%) had vaginal deliveries and 5 (9%) had caesarean sections. Majority of subjects primed with FC only were nulliparous (21/36, 58%) and of them majority (18/21, 86%) delivered vaginally. Out of 21 nullipara primed with Foley only, 15 (15/21, 71%) were uncomplicated pregnancies who have completed 41 weeks of gestation. From these 15, 13 delivered vaginally. In contrast, rest of the 20 (36%) subjects had FC followed by additional vaginal Prostaglandin E2 insertion. Thirteen of them (13/20, 65%) had vaginal deliveries. Of the 24 subjects who were induced due to completed 41 weeks of gestation with otherwise uncomplicated pregnancies, 17 (71%) had post-priming MBS of 6 or more with FC alone.

**Table 2:** General outcomes of the audit

Parity	Cervical priming with Foley catheter alone (n = 36) n (%)	Total vaginal deliveries including instrumental deliveries (with Foley catheter and Prostaglandin vaginal tablets) (n = 44) n (%)	Number of vaginal deliveries in Foley catheter only cases (n = 31) n (%)	Average duration of Foley catheter-in situ in hours (SD)	Average discomfort score according to 1–10 visual analogue scale (SD)
Parity-0	21 (58)	26 (59)	18 (58)	29.8 (16)	1.7 (1)
Parity-1	9 (25)	11 (25)	7 (23)	29.5 (18)	2.1 (2)
Parity-2	4 (11)	4 (9)	4 (13)	39.4 (9)	1.0 (0)
Parity-3	1 (3)	2 (5)	1 (3)	38.7 (14)	1.7 (1)
Parity-4	1 (3)	1 (2)	1 (3)	23.0 (0)	1.0 (0)

*SD* standard deviation

Relationship of mode of delivery with other parameters is summarized in Table 3. Twenty out of 24 (83%) women who completed 41 weeks of gestation with otherwise uncomplicated pregnancies delivered vaginally including single forceps delivery. Total vaginal delivery rate is 77% (43/56). There were 12 (12/56, 21%) caesarean sections. Having a MBS of 6 or more was related to the duration of Foley in situ (Fisher’s exact test, P = 0.05). During IOL 38 (67%) had no discomfort and 15 (26%) had only mild discomfort (Table 4). FC was deflated due to moderate discomfort in three women at 48 h of insertion and they had MBS of more than 6 at the time of deflation and they have been included in the analysis.

**Table 3:** Mode of delivery with other parameters

	Mode of delivery (n = 56)						
	Normal vaginal delivery	Forceps	Caesarean section due to lack of progression of labour	Caesarean section due to foetal distress	Caesarean section due to failed induction of labour	Total number of caesarean sections	Getting a caesarean section
Parity							Being nulliparous
Nulliparous (n = 32)	25	1	2	2	2	6	RR = 0.75 95% CI = 0.28–2.04
Multiparous	18	0	2	2	2	6	P = 0.81
Indication							Having an UP
UP (n = 24)^a^	19	1	2	1	1	4	RR = 0.6
GDM	18	0	2	1	2	6	95% CI = 0.21–1.70
FGR	5	0	0	1	0	1	P = 0.49
Reduced AFI	1	0	0	0	0	0	
Past section	0	0	0	0	1	1	
For comparison							Having Foley only
Women who had Foley only (n = 36)	30	1	2	2	1	5	RR = 0.40 95% CI = 0.15–1.09
Women who had Foley, then prostaglandin	13	0	2	2	3	7	P = 0.09

UP-Women who have completed 41 weeks of gestation with otherwise uncomplicated

*RR* relative risk, *95% CI* 95% confidence interval, pregnancies, *GDM* gestational Diabetes mellitus, *FGR* fetal growth restriction, *AFI* amniotic fluid index, *Past section* past history of caesarean section

^a^Relative risk assessed for this indication only

**Table 4:** Patient acceptability of Foley catheter as assessed with visual analogue scale (0–10)

Degree of discomfort (n = 56)	Frequency (%)
No discomfort	38 (67)
Mild discomfort	15 (26)
Moderate discomfort	3 (5)
Severe discomfort	0
Subjects with mild or no discomfort (≤3 score in visual analogue scale) (n = 53)	
Parity	
Nulliparous	30 (58)
Multiparous	23 (42)
Mode of delivery	
Vaginal or forceps delivery	43 (81)
Caesarean Section	10 (19)

## Discussion

As shown in the above results, subjects who were primed with FC followed by additional vaginal prostaglandins if needed after 48 h, mostly delivered vaginally (65%). Our unit has an IOL rate of 11%, which is way below the national average but comparable to the rates reported by others [2]. Careful management strategies adhering to guidelines as an academic unit might have resulted this low IOL rate. Many studies have reported that both FC and prostaglandin E2 gel are equally effective in pre induction cervical ripening [8, 15]. FC is a safe method of labor induction for the mother, fetus and newborn [16].

In Sri Lanka conventionally most of the Obstetric Units are practicing IOL at 41 weeks of gestation for women with otherwise uncomplicated pregnancies. Latest guideline on IOL published by SLCOG has mentioned that IOL is recommended for otherwise uncomplicated, low-risk women who are known with certainty to have reached 41 weeks of gestation [14]. Furthermore, this guideline elaborates that it is good practice to assess fetal wellbeing around 40 weeks to select women for conservative management until 41 weeks gestation [14]. This was later challenged because firstly, it was based on epidemiological data and secondly, it was calculated from the statistical distribution of the timing of delivery from the last menstrual period (LMP) [17]. Moreover, this definition did not consider the risk of pregnancy complications including stillbirth during late gestational ages. It is now accepted that gestational age assessment by LMP is not accurate [18]. Maturity of 39 week Asian fetuses may be equal to that of a 41 week Caucasian fetus, implying that Asian fetuses mature sooner than Caucasians [17]. South Asian and black women have shorter length of gestation compared to Caucasian indicating the likelihood of high early perinatal complications in south Asian and black women [17].

FC is much cheaper when compared to vaginal prostaglandin tablets. A FC costs 90 LKR (0.7 USD), while 3 mg of prostaglandin costs about 1500 LKR (11.5 USD). Therefore it seems to be a cost effective method in developing countries like Sri Lanka. It has also shown to be a safe method in cervical priming and found to have same efficacy when compared with prostaglandins [7, 10]. Some studies from developing countries on IOL have attempted to find out an economically feasible method as a cervical priming agent. Recently conducted PROBAAT trial in Netherlands, has evaluated cost-effectiveness of IOL at term with a FC compared to vaginal prostaglandin E2 gel [19]. The FC group showed higher costs due to longer labour ward occupation and less cost related to induction material and neonatal admissions [19]. However, FC usage has showed a comparable caesarean section rate compared with prostaglandin induction and therefore the incremental cost-effectiveness ratio has not been informative [19]. FC use resulted in fewer neonatal admissions and asphyxia/postpartum haemorrhage compared with prostaglandin use [19]. They have concluded that FC and prostaglandin E2 gel labour induction generate comparable costs [19]. Interestingly, an Australian trial had reported different results which are more in favour of FC as a better cost-effective method. In that study the only difference in cost between the three groups (Foley, double balloon catheter and prostaglandin E2) relate to the cost of the cervical ripening device as there were no differences between groups in length of time in labour ward, mode of delivery, postnatal complications, duration of hospital admission or re-presentation to hospital after discharge [7]. The cost of ripening devices used in the trial were substantially lower for the Foley catheter (AUS$2.00) compared with the double balloon catheter (AUS$81) and prostaglandin E2 gel (AUS$124 for two doses) [7]. In a setting where cost of labour ward stay is relatively less due to cheaper labour cost, FC seems to be a cost effective solution for cervical priming. A study from India concluded that vaginal misoprostol is a cheap, highly effective, stable at room temperature and easy to administer agent for labor induction [11]. They have shown that misoprostol is superior to FC/oxytocin [11]. However this result is debatable. A metaanalysis reported that vaginally administered misoprostol was more effective than dinoprostone vaginal insert for cervical priming and IOL and the safety profile of both drugs were similar [20]. This indicates that both FC and misoprostol has some economic advantages over prostaglandin. Although misoprostol is widely used worldwide for various indications in pregnancy, in Sri Lanka, it is not licensed at present [20]. Therefore FC becomes more important in IOL cost reduction in our setting.

As shown in above results, subjects who were primed with FC followed by additional vaginal prostaglandins after 48 h, had a high chance of getting a vaginal delivery (65%). We had to deflate FC at 48 h in 16 subjects (29%) with a total vaginal delivery rate of 79%. Ekele and Isah reported that most women (95%) would have expelled FC spontaneously within 72 h of insertion and a 91% vaginal delivery rate [21]. According to our results nulliparous women reported 25/32, 78% vaginal delivery rate. Study done in Australia among nulliparous women had reported 45/110, 41% of spontaneous vaginal delivery rate [7]. Nevertheless, as a conclusion of this trial they have mentioned that labour induction in nullipara with unfavourable cervices results in high caesarean delivery rates [7]. Although all methods (double balloon, single balloon and prostaglandin) in this study had similar efficacy, the single balloon catheter had offered the best combination of safety and patient comfort [7]. In our study, subjects who have completed 41 weeks of gestation with otherwise uncomplicated pregnancies and who were primed with FC alone (21/32) had reported 83% rate of vaginal delivery. Amongst who have completed 41 weeks, there were 15 nulliparous women reporting 13/15, 87% of vaginal delivery rate. This indicates that FC is a good option for the subjects with completed 41 weeks and especially nulliparous women in our unit.

However, a recent retrospective cohort study comparing nulliparous women with uncomplicated post term pregnancies with FC induction versus spontaneous labour has shown that Foley induction resulted in a sixfold increase in risk of caesarean section rate (odds ratio 6.2) [22]. But among parous women it was low and not significant [22]. In our study, we did not have a control group to compare. A recent Sri Lankan trial conducted among women with uncomplicated singleton pregnancies with 40 weeks and 6 days, has also shown that intracervical FC for 24 h was better than two doses of 25 μg misoprostol administered orally 4 h apart, for pre induction cervical ripening in these prolonged pregnancies [23]. There FC has shown to be effective for both nulliparous and multiparous giving higher MBS and lower caesarean section rate. In our study, subjects who needed Foley only have a lesser chance of getting a caesarean section compared to those subjects who needed Foley followed by prostaglandin (relative risk = 0.40, 95% CI = 0.15–1.09, P = 0.09). Although this is not statistically significant, the trend seen is biologically plausible, and might be confirmed with a larger sample size and greater statistical power.

Overall 53/56, 95% had mild or no discomfort with FC cervical priming indicating that FC has a good patient satisfaction. The only available study reporting patient satisfaction for FC using visual analogue scales (0–10) has shown that FC had best patient comfort during insertion and ripening phase both [7]. In this study prostaglandin and FC had similar pain scores during insertion whereas during ripening phase FC had greater patient comfort than prostaglandins (pain score > 4, 36% in FC group vs 63% in prostaglandin group, P < 0.001) [7].

## Conclusions

FC insertion is an effective method in cervical priming for IOL in our setting. It can be recommended for low/medium resource settings in developing countries as FC insertion is potentially cheaper, gives a good patient comfort and also a high chance of vaginal delivery rate. FC alone could be an effective method of IOL for the subjects who have completed 41 weeks of gestation with otherwise uncomplicated pregnancies.

Cost-effectiveness in low resource settings and pregnant women satisfaction with FC has to be better evaluated by further well controlled trials.

This study did not assess the risk of infection. A relatively small sample size and being a single arm study might have an impact on results. There is no comparison with another method.

## Abbreviations

IOL: induction of labour
DSHW: De Soysa Hospital for Women
MBS: Modified Bishop Score
LKR: Sri Lankan Rupees
USD: United States’ dollars
FC: Foley catheter
